# Bipolar Hemiarthroplasty should not be selected as the primary option for intertrochanteric fractures in elderly patients

**DOI:** 10.1038/s41598-020-61387-3

**Published:** 2020-03-16

**Authors:** Jincheng Huang, Yanxin Shi, Weiyu Pan, Zhen Wang, Yonghui Dong, Yu Bai, Aiguo Wang, Yongqiang Zhao, Jia Zheng, Hongkai Lian

**Affiliations:** 1 Department of Orthopedics, Henan Provincial People’s Hospital, People’s Hospital of Zhengzhou University, People’s Hospital of Henan University, Zhengzhou, Henan 450003 P.R. China; 2 Department of Orthopedics, Zhengzhou Orthopedics Hospital, Zhengzhou, Henan 450052 P.R. China; 3 Department of Orthopedics, Zhengzhou Central Hospital, Zhengzhou, Henan 450007 P.R. China

**Keywords:** Clinical trials, Trauma

## Abstract

Intertrochanteric fractures (ITFs) in the elderly are still a big challenge for clinical doctors. Although proximal femoral nail antirotation (PFNA) and bipolar hemiarthroplasty (BPH) are selected by most of the orthopaedic surgeons for elderly ITFs patients, there is still no consensus on the superiority of PFNA and BPH for ITFs in elderly. In this study, we hypothesized that BPH should not be selected as the primary option for ITFs in elderly patients, and analyzed clinical data of 202 elderly ITFs patients aged 80 years or more treated with PFNA (Group A) and BPH (Group B) to compare the early outcome of PFNA and BPH for ITFs in elderly patients aged 80 years or more. We found that operation time and blood loss during surgery in group A are less than in Group B. Time of weight bearing after operation in Group A is longer than in Group B. Incidence of complications 2 weeks after operation in Group A is 9.29% less than 25.81% in Group B (χ^2^ = 9.539, p = 0.002). Mortality rates 12 months after operation in Group A is 11.43% similar with 19.35% in Group B (χ^2^ = 2.261, p = 0.133). Harris Hip Score 12 months after operation in Group A is 68.00 ± 29.11 points similar with 65.73 ± 33.29 points in Group B (t = 0.490, p = 0.625). Therefore, for elderly ITFs patients aged 80 years or more, BPH should not be selected as the primary option for ITFs in elderly patients.

## Introduction

Intertrochanteric fractures (ITFs), commonly occurred in the elderly, are still a big challenge for orthopaedic surgeons due to the multitude of co-morbidities and high 1-year mortality rate associated with them^1,2^. In order to reduce disability and mortality rate, early surgical procedure, which allows early mobilization, restores the function of the limb, has become the general consensus for the ITFs treatment in the literature^3^. Different from the various operation methods for young ITFs patients (Dynamic hip screws (DHS), Medoff sliding plate (MSP), Percutaneous compression plating (PCCP), Less invasive stabilization system (LISS), Gamma nail and PFNA), PFNA and BPH are now the two main methods used by surgeons for ITFs in the elderly^4,5^. However, until now, there is still no consensus on the superiority of PFNA and BPH for the elderly ITFs patients^6^. As a result, we decide to explore which one (PFNA or BPH) is better for the elderly ITFs patients especially those aged 80 years or more.

## Materials and Methods

### Subjects

**Figure 1:**
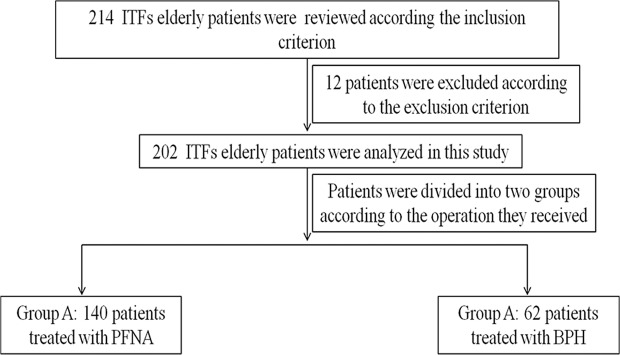
Flow chart of patients in this study.

This retrospective study was approved by the Ethics Committee of Zhengzhou Central Hospital and performed in accordance with the Helsinki Declaration. Clinical data of 202 ITFs elderly patients (≥80 years old) treated with PFNA and BPH within 3 weeks after injury from department of Orthopedics in Henan Provincial People’s Hospital, Zhengzhou Orthopedics Hospital, and Zhengzhou Central from January 1^st^ 2017 to July 31^th^ 2018 were retrospectively analyzed. Exclusion criterion includes: patients with 1, pathologic fractures; 2, concomitant pelvic fracture; 3, fractures associated with polytrauma; 4, immobility or walking difficulties before fracture; 5, infection in the hip or pelvic area or sepsis; 6, preexisting ipsilateral femoral implant; 7, mental illness or acute confusion without a history of dementia; 8, preoperative ASA physical status: grade IV; 9, duration of follow-up less than 12 months; 10, malignant tumors. Flow chart of patients in this study is shown in Fig. 1.

Patients enrolled in this study were grouped as follows: Group A: 140 patients treated with PFNA; Group B: 62 patients treated with BPH. Record gender, age, period of follow-up, fracture classification(according to Evans-Jensen), preoperative ASA physical status, interval between injury and operation, method of anaesthesia, duration of operation time, blood loss during surgery, time of weight bearing after operation, incidence of complications 2 weeks after operation, mortality rates and Harris Hip Score 12 months after operation.

### Surgical techniques

Operations were performed under spinal anaesthesia or general anaesthesia.

BPH was performed through lateral approach with the patient in a lateral decubitus position and the affected hip was uppermost. First, fracture fragments including the femoral head, neck, calcar (posteromedial fragment) and lesser trochanter were removed; Second, femoral canal was prepared using a broach and a Wagner SL cementless distal fixation femoral stem (Zimmer, USA) was inserted into the femoral canal; Third, displaced greater trochanter fracture fragments were reduced and fixed by wire as a ‘8’ shape; Fourth, trial reduction was performed and appropriate neck length and bipolar head diameter were selected; Fifth, reattach the capsule and close the wound in layers.

PFNA (Synthes; USA) was performed on traction tables in a supine position under C-arm fluoroscopy. First, perform the closed reduction of the fracture fragments; Second, insert the nail from the lateral aspect of the greater trochanter; Third, insert the column screw until its tip as close as 5 mm to the subchondral bone; Forth, fix the locking bolt and the end cap; Seventh, close the wound in layers.

### Peri-operative protocol

Antibiotic prophylaxis was used within 30–60 min before incision and within the first 24 h postoperatively in the two groups. Low molecular weight heparin was used daily and continued until check out. Aspirin was used after checking out for another one month.

For the BPH group, patients were permitted weight bear standing in the first week after surgery depending on the physical status of the patients and encouraged to use a walker until the patients had adequate muscle strength and balance. Excessive hip flexion (>90°) and adduction were not allowed within the first six weeks after surgery.

For the PFNA group, patients were encouraged to sit halfway and exercise lower extremities in bed on the first day after operation. Time of weight bearing standing was decided depending on the reduction of bone structures and the position of the fixation.

Patients were followed up at 6 weeks, 3 months, half an year, 1 year, and annually thereafter for clinical and radiological examinations. If the patient can’t come to our department personally, the clinical outcomes were evaluated by telephone, and the radiological outcomes were evaluated by X-ray films which obtained at their local hospitals.

### Statistical analysis

Quantitative data were expressed as mean ± standard deviation, and counting data are presented as percentage. t-test was used for the comparison of measurement data, while Chi-square test (χ^2^) was used to compare the counting data among groups. P value less than 0.05 was considered as significant difference. All statistical analyses were performed using IBM SPSS Statistics (version 19, IBM SPSS Software).

## Results

### Comparison of basic clinical data between the two groups before operation

A total of 214 ITFs patients were reviewed, 12 patients were excluded for there were three pathologic fractures, three patients with walking difficulties before fracture, two fractures associated with polytrauma and four patients were lost due to failed followed up. Finally, 202 patients were followed up successfully. Patient demographics are presented in Fig. 1. As shown in Table 1, there were no significant differences between the groups in terms of gender, age, fracture classification according to Evans- Jensen, follow-up duration, pre-operative ASA physical status classification, method of anaesthesia and interval between injury and operation.

**Table 1:** Comparison of general data between patients from BPH and PFNA group.

Group	Age (year)	Gender		Evans-Jesen classification					Follow-up duration(month)	Pre-operative ASA classification		Method of anaesthesia		Interval between injury and operation(day)
		Male	Female	I	II	III	IV	V		2	3	Intraspinal anesthesia	General anesthesia	
A	86.06 ± 4.16	37	103	11	10	46	7	66	20.04 ± 8.37	49	91	129	11	7.91 ± 5.53
B	86.19 ± 4.75	16	46	5	5	11	9	32	17.41 ± 8.04	21	41	55	7	7.05 ± 4.64
Statistic	t = −0.195 p = 0.846	χ^2^ = 0.009 p = 0.926		χ^2^ = 8.484 p = 0.075					t = 2.082 p = 0.039	χ^2^ = 0.024 p = 0.876		χ^2^ = 0.624 p = 0.430		t = 1.076 p = 0.283

### Comparison of operative statistics between the two groups

When compare operative statistics (Table 2) such as duration of operation time (103.47 ± 41.09 min in Group A and 119.26 ± 32.32 min in Group B), blood loss during surgery (71.50 ± 26.09 ml in Group A and 187.90 ± 98.22 ml in Group B) between patients from the two groups, the difference are significant.

**Table 2:** Comparison of duration of operation time, blood loss during surgery, time of weight bearing after operation, incidence of bad complications two weeks after operation, mortality rate and Harris Hip Score 12 months after operation between patients from Group A and B.

Group	Duration of operation time(min)	Blood loss during surgery(ml)	Time of weight bearing after operation(day)	Incidence of bad complications two weeks after operation(percentage)	Mortality rate 12 months after operation(percentage)	Harris Hip Score 12 months after operation(points)
A	103.47 ± 41.09	71.50 ± 26.09	47.96 ± 31.16	9.29%(13/140)	11.43%(16/140)	68.00 ± 29.11
B	119.26 ± 32.32	187.90 ± 98.22	4.95 ± 2.25	25.81%(16/62)	19.35%(12/62)	65.73 ± 33.29
Statistic	t = −2.680	t = −13.057	t = 8.567	χ^2^ = 9.539 p = 0.002	χ^2^ = 6.603	t = 0.490
	p = 0.008	p = 0.000	p = 0.000		p = 0.010	p = 0.625

### Comparison of postoperative data between the two groups

When compare the postoperative data such as time of weight bearing after operation (47.96 ± 31.16 days in Group A is longer than 10.68 ± 21.36 days in Group B), incidence of bad complications 2 weeks after operation (Table 3), 13 patients (9.29%) in Group A is much less than 16 patients (25.81%) in Group B, the difference are significant. However, functional outcome (Harris Hip Score 12 months after operation) in Group A (68.00 ± 29.11 points) is similar with Group B (65.73 ± 33.29 points), and mortality rates 12 months after operation in Group A is 11.43% similar with 19.35% in Group B, the difference are not significant.

**Table 3:** Bad complications 2 weeks after operation in patients from Group A and Group B.

Complications	Group A	Group B
Pulmonary infection	5	7
Urinary infection	1	
Gastrointestinal dysfunction	5	2
Cut through of screws	1	
Acute cerebral infarction		3
Heart failure	1	3
Cholangitis		1

## Discussion

Due to the aging of the population and rapid development of society, the number of elderly patients with ITFs is increasing year by year. Open or closed reduction with internal fixation has been accepted as effective treatments for this injury^7^. An ideal surgical technique for elderly ITFs patients should have the least intra and post operative morbidity^8^. Although proximal femoral nail antirotation (PFNA) has been selected by most surgeons for elderly ITFs patients^9–11^, failures of PFNA have also been reported due to extensive comminution, osteoporosis or long bedridden duration^11^. As a result, BPH, which permits early full-weight bearing, avoids the failures of osteosynthesis, was first used in 1978 and subsequently used by other surgeons for ITFs treatment with satisfied results^12^, and has been suggested as an alternative method for elderly ITFs patients^5,13^. Unfortunately, researchers also found that BPH brings much more surgical injury than PFNA to patients due to the longer operation time and much more blood loss, and recommended that BPH should be undertaken with caution in carefully selected patients^14,15^. However, it is still unclear whether BPH or PFNA is the better choice for elderly ITFs patients. In this study, we found that although BPH and PFNA have similar functional outcome and mortality rates 12 months after operation, BPH has more postoperative complications in elderly patients with ITFs, BPH is not a good primary treatment for TIFs in elderly patients.

The goals of treatment of ITFs in the elderly are to regain preoperative ambulatory status with the lowest rate of medical and surgical complications^16^. Similar with published data^14^, in this study, we also find that PFNA and BPH have similar functional outcome, which means in the term of functional recovery, either PFNA or BPH is accepted for elderly ITFs patients. Consistent with previous results^17,18^, we also found that PFNA has shorter duration of operation time and less blood loss during surgery than BPH, which implies that PFNA does less surgical injury to patients. However, different from the hypothesis that longer bed-ridden (time of weight bearing after operation in Group A is 47.96 ± 31.16 days longer than 10.68 ± 21.36 days in Group B) leads to a high rate of general complications, our data demonstrated that incidence of bad complications two weeks after operation in Group A is much lower than Group B. For this phenomenon, we think there are at least three reasons, first, for the elderly patients underwent surgical treatment, surgical treatment itself is the second trauma to the patients, so less surgical trauma (PFNA) will bring less post-operative complications. Second, although the time of weight bearing after operation in Group A is much longer than in Group B, patients in Group A could exercise their lower extremities and sit halfway in bed on the first day after operation, which is totally different from the preoperatively unable to exercise due to pain. Third, although patients in Group B have early time of weight bearing after operation, due to the physical and psychological injury by the fracture, they dare not exercise as young patients to avoid falling down again and just stand around the bed, flex and extend knee and hip joints mildly. So, to some extent, the benefit of “early exercise” in Group B is similar with “bed-ridden exercise” in Group A.

Although PFNA does less surgical injury to patients, there are no significant difference when compare the mortality rates between patients from the two groups 12 months after operation. The underlying reason may be that only elderly ITFs patients aged 80 years or more were included (mean age was 86.10 years in our study), whose remaining life expectancy is short even though they do not suffer from the ITFs and PFNA or BPH.

Overall, in this study, we found that although BPH and PFNA have similar functional outcome and mortality rates 12 months after operation, BPH has more postoperative complications in elderly patients with ITFs, BPH is not a good primary treatment for TIFs in elderly patients. But there are some limitations in this study, first, our study was a retrospective controlled study, although the patient groups appeared similar, patients were not randomly assigned to the groups. Second, the duration of follow-up is short. However, a long-term follow-up has little clinical relevance considering the remaining life expectancy.
